# Nomogram based on pan-immune-inflammation value to predict short-term prognosis in spontaneous intracerebral hemorrhage

**DOI:** 10.3389/fneur.2025.1606436

**Published:** 2025-08-12

**Authors:** Shuai Wang, Wei Zhang, Jingjing Li, Xinxin Yang, Yuqiao Wang

**Affiliations:** ^1^Department of Neurology, The First Clinical Medical College of Xuzhou Medical University, Xuzhou, China; ^2^Department of Neurology, Affiliated Hospital of Xuzhou Medical University, Xuzhou, China; ^3^Department of Neurology, Affiliated Brain Hospital of Nanjing Medical University, Nanjing, China

**Keywords:** intracerebral hemorrhage, nomogram, pan-immune-inflammation value, clinical study, prognosis

## Abstract

**Introduction:**

The aim of this study was to investigate the impact of the Pan-Immune-Inflammation Value (PIV) on the prognosis of spontaneous intracerebral hemorrhage (ICH) and to develop and validate a nomogram for identifying patients with a poor prognosis following ICH.

**Methods:**

We retrospectively collected the clinical data of 742 patients with ICH admitted to the Affiliated Hospital of Xuzhou Medical University from September 2018 to March 2024. A modified Rankin Scale score > 3 at 90 days after discharge was defined as a poor short-term prognosis. The enrolled patients were randomly assigned to a training cohort and a validation cohort in a 7:3 ratio. In the training cohort, risk factors associated with poor short-term prognosis were identified through univariate and multivariate logistic regression analyses. Based on these risk factors, a nomogram was developed and validated.

**Results:**

Of the 742 ICH patients included in this study, 519 were assigned to the training cohort and 223 to the validation cohort. Multivariate logistic regression analysis identified several risk factors for poor prognosis of ICH: brainstem hemorrhage (OR = 3.17, 95% CI = 1.80–5.59, *p* < 0.01), reduced activated partial thromboplastin time (APTT) (OR = 0.94, 95% CI = 0.89–0.99, *p* = 0.047), large bleeding volume (OR = 1.06, 95% CI = 1.04–1.09, *p* < 0.01), low Glasgow Coma Scale (GCS) score (OR = 0.76, 95% CI = 0.70–0.82, *p* < 0.01), and high PIV level (OR = 1.01, 95% CI = 1.01–1.01, *p* < 0.01). A nomogram was constructed based on these factors. The area under the receiver operating characteristic curve was 0.86, indicating good discrimination ability. The Hosmer-Lemeshow goodness-of-fit test for the validation cohort demonstrated that the model had satisfactory calibration. Decision curve analysis revealed that the nomogram had clinical utility across a wide range of threshold probabilities.

**Conclusion:**

A high PIV level, large bleeding volume, and low GCS score are significant risk factors for poor prognosis in patients with ICH. The nomogram based on these factors demonstrates robust predictive performance.

## 1 Introduction

Intracerebral hemorrhage (ICH) is defined as a non-traumatic rupture of intracerebral blood vessels, leading to the accumulation of blood within the brain parenchyma. The incidence of ICH is estimated at 12–15 cases per 100,000 person-years. ICH accounts for approximately 15% of all strokes and 10–30% of all stroke-related hospitalizations in Western countries. In contrast, the proportion of ICH in China is significantly higher, ranging from 18.8 to 47.6% of all stroke cases. ICH is highly lethal, with a 30-day mortality rate as high as 35–52%, imposing a substantial burden on society and families (1).

Early and accurate identification of the severity and prognosis of ICH is crucial for improving clinical outcomes. Several predictors of poor prognosis have been identified, including the Glasgow Coma Scale (GCS) score at admission, hemorrhage location and volume, the presence of a mixed sign on computed tomography (CT), midline shift, elevated blood glucose levels, and increased neutrophil counts (2–8). However, clinical scoring systems such as the GCS primarily rely on subjective clinical assessments, which may be subject to interobserver variability. Additionally, imaging-based evaluations, while informative, are often static and do not easily facilitate dynamic monitoring of the patient’s condition. Therefore, there is a need to identify objective and readily accessible laboratory biomarkers that can provide a more reliable and dynamic assessment of the patient’s status.

A substantial body of evidence indicates that the inflammatory response plays a pivotal role in secondary brain injury following ICH. After ICH, various blood components rapidly infiltrate the brain parenchyma, activating microglia and prompting the release of inflammatory cytokines and chemokines. These mediators recruit peripheral immune cells to the site of injury, where infiltrating leukocytes release proinflammatory and cytotoxic factors that increase capillary permeability, promote cellular swelling, disrupt the blood–brain barrier (BBB), and exacerbate cerebral edema (9, 10). Clinical studies have demonstrated that early peripheral neutrophilia serves as a marker of post-ICH inflammation and is associated with early neurological deterioration and poor prognosis (11). Conversely, lymphopenia has been linked to an increased risk of complications and worse prognosis in ICH patients (12). However, individual biomarkers are often unstable and may not reliably reflect the complex pathophysiology of ICH. Recent studies have shown that composite inflammatory indicators, which integrate multiple parameters, offer greater stability and potentially higher predictive value. The Pan-Immune-Inflammation Value (PIV) is calculated from the peripheral blood counts of neutrophils, monocytes, platelets, and lymphocytes using the formula: PIV = [neutrophil count (10^9^/L) × monocyte count (10^9^/L) × platelet count (10^9^/L)] / lymphocyte count (10^9^/L). This value provides a comprehensive reflection of the systemic inflammatory response and immune imbalance. As a composite of multiple immune cell parameters, PIV can more accurately capture the overall inflammatory status, offering a more robust tool for prognostic assessment (13). Previous studies have identified an association between PIV and ischemic stroke. Moreover, PIV has been shown to predict the short-term prognosis of patients with acute cerebral infarction following intravenous thrombolysis (14). Elevated PIV levels may indicate secondary immunosuppression and increase the risk of post-stroke pneumonia. However, the association between PIV and ICH has been less extensively studied, and it remains unclear whether PIV can serve as a predictor of poor prognosis in ICH patients. The objective of this study was to investigate the relationship between PIV and poor prognosis in ICH patients and to develop and validate a nomogram incorporating PIV and other risk factors, which may facilitate early identification of patients’ conditions in clinical practice.

## 2 Materials and methods

### 2.1 Patients

A total of 742 patients with ICH who were hospitalized at the Affiliated Hospital of Xuzhou Medical University from September 2018 to March 2024 were retrospectively included as study subjects. The inclusion criteria were as follows: (1) meeting the diagnostic criteria outlined in the *Chinese Guidelines for Diagnosis and Treatment of Acute Intracerebral Hemorrhage 2019*; (2) confirmation of ICH diagnosis via CT within 24 h of admission; (3) no prior receipt of specialized treatment, such as surgery or intensive care, from other hospitals before admission. The exclusion criteria were as follows: (1) Incomplete clinical data, including imaging, laboratory results, and patients who were lost to follow-up; (2) patients with systemic diseases, including severe hepatic and renal dysfunction, hematologic disorders, malignancies, as well as those who had infections before admission, used antibiotics or immunosuppressive agents within 1 week before admission, or had immune system diseases; (3) ICH resulting from trauma, aneurysm rupture, cerebral vascular malformation, cerebral amyloidosis, or other causes; (4) transformation from cerebral infarction to hemorrhage; (5) aged < 18 years. This study was approved by the Ethics Committee of the Affiliated Hospital of Xuzhou Medical University (grant no. XYFY2024-KL346-01).

### 2.2 Clinical characteristics data

We collected demographic and clinical characteristics, including age, gender, history of smoking, alcohol consumption, hypertension, diabetes mellitus, coronary heart disease, cerebral infarction, previous ICH, and body mass index (BMI). Additionally, we recorded the patients’ GCS scores on admission (range 0–15; lower scores indicate more severe disorders of consciousness), bleeding site (categorized as brainstem or lobar based on the primary hemorrhagic site), Laboratory data within 24 h of admission were also collected, including neutrophil count, lymphocyte count, monocyte count, platelet count, albumin level, blood glucose level, and activated partial thromboplastin time (APTT). All patients were followed up for 3 months after the onset of the disease, and the modified Rankin Scale (mRS) score was used to assess prognosis. Patients were classified into two groups based on their mRS score: good prognosis (mRS score 0–3) and poor prognosis (mRS score 4–6). PIV was calculated using the following formula: PIV = (neutrophil count × platelet count × monocyte count) / lymphocyte count.

### 2.3 Statistical analysis

Data analysis was performed using R version 4.1.3. Normally distributed continuous data are presented as mean ± standard deviation (X ± SD), and comparisons between groups were made using the *t*-test. Non-normally distributed continuous data are presented as median and interquartile range [M (IQR)], and comparisons between groups were made using the Mann–Whitney U test. Categorical data are presented as numbers (%), and comparisons between groups were made using the chi-square (*χ*^2^) test. Univariate Logistic regression analysis was used to identify potential risk factors for poor prognosis of ICH in the training cohort. Multivariate Logistic regression analysis was then used to further identify independent risk factors and to establish a nomogram prediction model. The discrimination of the model was evaluated using receiver operating characteristic (ROC) curves. Calibration curves and Hosmer-Lemeshow (H-L) goodness-of-fit tests were used to assess the accuracy of the model, and decision curve analysis (DCA) was used to evaluate its clinical effectiveness. A *p* < 0.05 was considered to indicate a statistically significant difference.

## 3 Results

**Figure 1:**
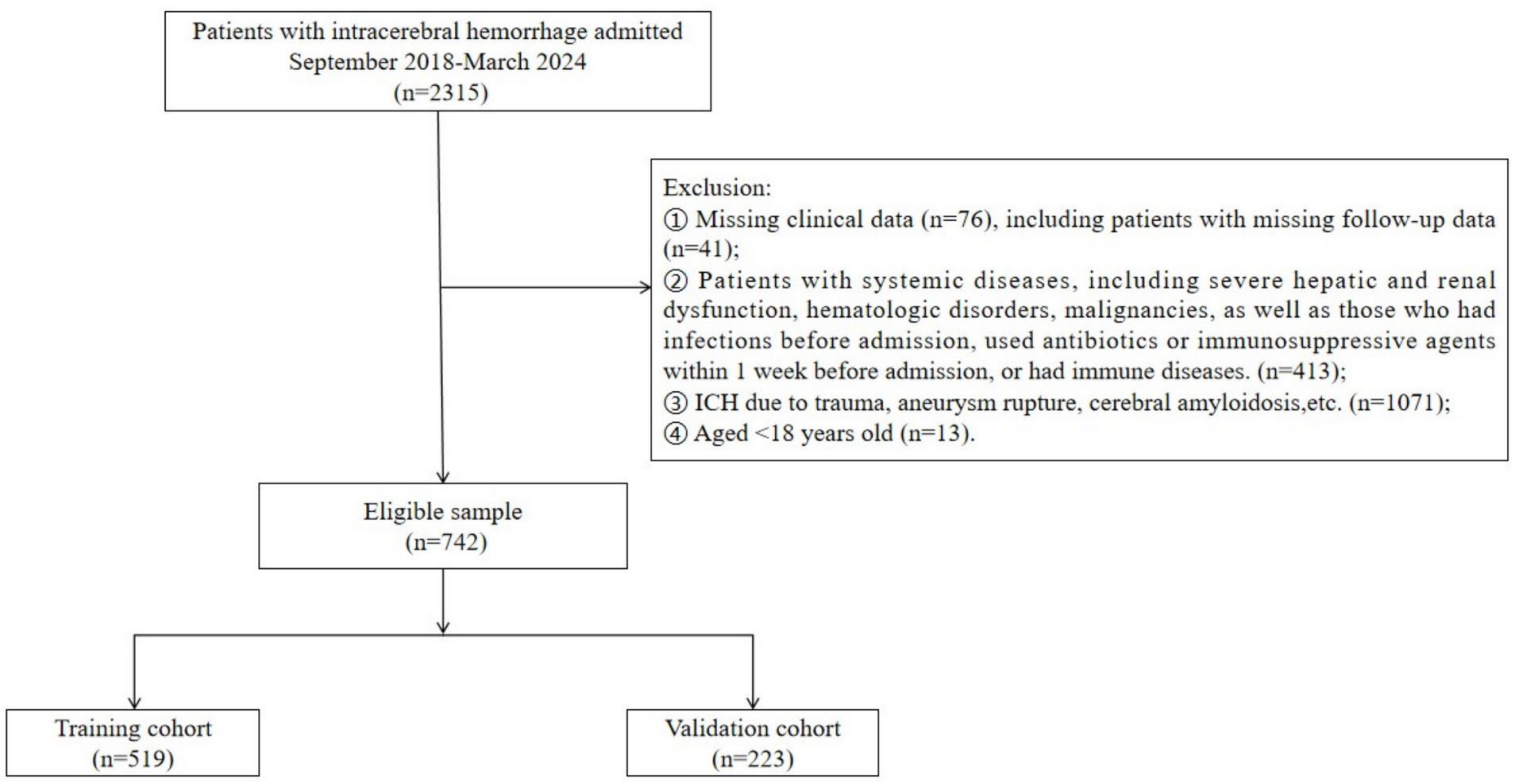
Flowchart of patients’ selection.

A total of 742 ICH patients were included in the study. Of these, 433 patients (58.36%) had a poor prognosis. The patients were randomly assigned to a training cohort (*n* = 519) and a validation cohort (*n* = 223) (Figure 1).

In the training cohort, comparisons of general data between the good prognosis group and the poor prognosis group revealed that the poor prognosis group had a higher prevalence of previous ICH, surgical treatment, and brainstem hemorrhage. Additionally, the poor prognosis group exhibited higher levels of leukocytes, neutrophils, monocytes, blood glucose, bleeding volume, and PIV at admission. Conversely, total cholesterol, low-density lipoprotein (LDL), activated partial thromboplastin time (APTT), and GCS score were lower in the poor prognosis group. All differences were statistically significant (*p* < 0.05) (Table 1).

**Table 1:** Comparison of general information between the good prognosis group and the poor prognosis group at the training cohort.

Variables	Total (*n* = 519)	Good prognosis group (*n* = 218)	Poor prognosis group (*n* = 301)	Statistic	*P*
Age, Mean ± SD	58.25 ± 12.86	57.78 ± 12.96	58.58 ± 12.80	*t* = −0.70	0.485
BMI, Mean ± SD	25.85 ± 4.13	26.14 ± 4.34	25.65 ± 3.97	*t* = 1.34	0.182
Leukocyte, Mean ± SD	10.25 ± 3.90	8.48 ± 2.79	11.53 ± 4.08	*t* = −10.09	**<0.001**
Neutrophil, Mean ± SD	8.03 ± 3.91	6.23 ± 2.88	9.34 ± 4.04	*t* = −10.25	**<0.001**
Lymphocyte, Mean ± SD	1.64 ± 1.14	1.70 ± 0.91	1.60 ± 1.27	*t* = 1.04	0.300
Monocyte, Mean ± SD	0.47 ± 0.22	0.42 ± 0.16	0.50 ± 0.24	*t* = −4.56	**<0.001**
Platelet, Mean ± SD	204.95 ± 59.77	202.24 ± 55.38	206.92 ± 62.78	*t* = −0.88	0.380
Hemoglobin, Mean ± SD	142.81 ± 17.35	143.31 ± 16.45	142.44 ± 17.98	*t* = 0.56	0.575
Albumin, Mean ± SD	42.93 ± 4.28	43.00 ± 3.95	42.87 ± 4.51	*t* = 0.34	0.736
Creatinine, Mean ± SD	63.69 ± 27.71	61.49 ± 23.02	65.28 ± 30.61	*t* = −1.61	0.108
Total cholesterol, Mean ± SD	4.22 ± 1.01	4.36 ± 1.02	4.12 ± 0.99	*t* = 2.63	**0.009**
Triglyceride, Mean ± SD	1.55 ± 1.17	1.60 ± 1.38	1.51 ± 0.98	*t* = 0.88	0.381
HDL, Mean ± SD	1.10 ± 0.34	1.11 ± 0.35	1.10 ± 0.34	*t* = 0.31	0.755
LDL, Mean ± SD	2.44 ± 0.82	2.52 ± 0.79	2.38 ± 0.84	*t* = 1.99	**0.048**
Prothrombin time, Mean ± SD	11.19 ± 2.16	11.30 ± 2.80	11.11 ± 1.52	*t* = 0.95	0.341
Percentage activity, Mean ± SD	102.47 ± 14.07	101.87 ± 14.32	102.90 ± 13.90	*t* = −0.82	0.413
PTR, Mean ± SD	0.99 ± 0.20	1.00 ± 0.26	0.98 ± 0.14	*t* = 1.31	0.189
INR, Mean ± SD	0.99 ± 0.20	1.00 ± 0.26	0.98 ± 0.15	*t* = 1.31	0.190
APTT, Mean ± SD	26.10 ± 3.80	26.87 ± 3.76	25.54 ± 3.74	*t* = 3.97	**<0.001**
Fibrinogen, Mean ± SD	2.75 ± 0.73	2.75 ± 0.70	2.75 ± 0.74	*t* = −0.03	0.979
TT, Mean ± SD	16.23 ± 1.44	16.18 ± 1.34	16.28 ± 1.51	*t* = −0.78	0.434
Glucose, Mean ± SD	7.76 ± 2.68	7.33 ± 2.81	8.08 ± 2.53	*t* = −3.20	**0.001**
HbA1c, Mean ± SD	6.19 ± 1.27	6.25 ± 1.41	6.14 ± 1.16	*t* = 0.95	0.341
Bleeding volume, Mean ± SD	15.98 ± 17.53	8.70 ± 11.51	21.26 ± 19.19	*t* = −9.28	**<0.001**
GCS, Mean ± SD	9.96 ± 3.64	11.96 ± 2.85	8.51 ± 3.47	*t* = 12.42	**<0.001**
PIV, Mean ± SD	692.28 ± 689.91	415.21 ± 426.39	892.95 ± 770.82	*t* = −9.02	**<0.001**
Male, *n* (%)	360 (69.36)	150 (68.81)	210 (69.77)	*χ*^2^ = 0.05	0.815
Smoking, *n* (%)	235 (45.28)	97 (44.50)	138 (45.85)	*χ*^2^ = 0.09	0.760
Alcohol drinking, *n* (%)	231 (44.51)	97 (44.50)	134 (44.52)	*χ*^2^ = 0.00	0.996
Hypertension, *n* (%)	396 (76.30)	165 (75.69)	231 (76.74)	*χ*^2^ = 0.08	0.780
Diabetes, *n* (%)	69 (13.29)	34 (15.60)	35 (11.63)	*χ*^2^ = 1.73	0.189
CHD, *n* (%)	46 (8.86)	20 (9.17)	26 (8.64)	*χ*^2^ = 0.05	0.832
Ischemic Stroke, *n* (%)	116 (22.35)	53 (24.31)	63 (20.93)	*χ*^2^ = 0.83	0.361
Prior ICH, *n* (%)	57 (10.98)	17 (7.80)	40 (13.29)	*χ*^2^ = 3.90	**0.048**
Surgery, *n* (%)	148 (28.52)	21 (9.63)	127 (42.19)	*χ*^2^ = 65.75	**<0.001**
BSH, *n* (%)	222 (42.77)	68 (31.19)	154 (51.16)	*χ*^2^ = 20.60	**<0.001**
Involving ventricles, *n* (%)	24 (4.62)	8 (3.67)	16 (5.32)	*χ*^2^ = 0.78	0.378

BMI, Body Mass Index; HDL, High-Density Lipoprotein; LDL, Low-Density Lipoprotein; PTR, Partial Thromboplastin Time; INR, International Normalized Ratio; APTT, Activated Partial Thromboplastin Time; TT, thrombin Time; HbA1c, Glycated Hemoglobin A1c; GCS, Glasgow Coma Scale; PIV, pan-immune-inflammation value; CHD, Coronary Heart Disease; ICH:intracerebral hemorrhage; BSH, Brainstem Hemorrhage.

Univariate logistic regression analysis revealed that brainstem hemorrhage, leukocytosis, neutrophilia, monocytosis, decreased total cholesterol, decreased LDL, surgery, decreased activated APTT, elevated blood glucose, large hemorrhage volume, low GCS score, and high PIV level were associated with poor prognosis in ICH patients (all *p* < 0.05). These variables were subsequently included in a multivariate logistic regression analysis. Given that neurosurgical intervention is typically considered only for patients with severe clinical conditions, this variable was excluded from the nomogram construction to minimize potential bias (Table 2). The results indicated that brainstem hemorrhage (OR = 3.17, 95% CI = 1.80–5.59, *p* < 0.01), decreased APTT (OR = 0.94, 95% CI = 0.89–0.99, *p* = 0.047), large bleeding volume (OR = 1.06, 95% CI = 1.04–1.09, *p* < 0.01), low GCS score (OR = 0.76, 95% CI = 0.70–0.82, *p* < 0.01), and high PIV level (OR = 1.01, 95% CI = 1.01–1.01, *p* < 0.01) were independent risk factors for poor prognosis in ICH patients (Table 3).

**Table 2:** Univariate logistic analysis of poor prognosis of ICH.

Variables	*β*	S. E	Z	*P*	OR (95%CI)
Age	0.00	0.01	0.70	0.484	1.00 (0.99–1.02)
BMI	−0.03	0.02	−1.33	0.183	0.97 (0.93–1.01)
Leukocyte	0.26	0.03	8.13	**<0.001**	1.29 (1.22–1.38)
Neutrophil	0.26	0.03	8.31	**<0.001**	1.30 (1.22–1.38)
Lymphocyte	−0.08	0.08	−1.03	0.301	0.92 (0.79–1.07)
Monocyte	1.91	0.46	4.12	**<0.001**	6.76 (2.72–16.78)
Platelet	0.00	0.00	0.88	0.379	1.00 (1.00–1.00)
Hemoglobin	−0.00	0.01	−0.56	0.575	1.00 (0.99–1.01)
Albumin	−0.01	0.02	−0.34	0.735	0.99 (0.95–1.03)
Creatinine	0.01	0.00	1.52	0.129	1.01 (1.00–1.01)
Total cholesterol	−0.23	0.09	−2.59	**0.010**	0.79 (0.66–0.94)
Triglyceride	−0.07	0.08	−0.87	0.385	0.94 (0.81–1.09)
HDL	−0.08	0.26	−0.31	0.754	0.92 (0.56–1.53)
LDL	−0.22	0.11	−1.97	**0.048**	0.81 (0.65–0.99)
Prothrombin time	−0.04	0.04	−0.91	0.361	0.96 (0.88–1.05)
Percentage activity	0.01	0.01	0.82	0.413	1.01 (0.99–1.02)
PTR	−0.63	0.51	−1.22	0.222	0.53 (0.20–1.46)
INR	−0.61	0.49	−1.23	0.220	0.55 (0.21–1.44)
APTT	−0.10	0.03	−3.72	**<0.001**	0.91 (0.86–0.95)
Fibrinogen	0.00	0.12	0.03	0.979	1.00 (0.79–1.28)
TT	0.05	0.06	0.78	0.433	1.05 (0.93–1.19)
Glucose	0.11	0.04	3.13	**0.002**	1.12 (1.04–1.20)
HbA1c	−0.07	0.07	−0.95	0.340	0.94 (0.82–1.07)
Bleeding volume	0.06	0.01	7.16	**<0.001**	1.07 (1.05–1.08)
GCS	−0.32	0.03	−9.73	**<0.001**	0.73 (0.68–0.77)
PIV	0.01	0.00	7.11	**<0.001**	1.01 (1.01–1.01)
Male	0.05	0.19	0.23	0.815	1.05 (0.72–1.53)
Smoking	0.05	0.18	0.31	0.760	1.06 (0.74–1.50)
Alcohol drinking	0.00	0.18	0.01	0.996	1.00 (0.70–1.42)
Hypertension	0.06	0.21	0.28	0.780	1.06 (0.70–1.60)
Diabetes	−0.34	0.26	−1.31	0.190	0.71 (0.43–1.18)
CHD	−0.07	0.31	−0.21	0.832	0.94 (0.51–1.72)
Ischemic Stroke	−0.19	0.21	−0.91	0.362	0.82 (0.54–1.25)
Prior ICH	0.59	0.30	1.95	0.051	1.81 (1.00–3.29)
Surgery	1.92	0.26	7.47	**<0.001**	6.85 (4.13–11.34)
BSH	0.84	0.19	4.50	**<0.001**	2.31 (1.60–3.33)
Involving ventricles	0.39	0.44	0.88	0.381	1.47 (0.62–3.51)

BMI, Body Mass Index; PTR, Partial Thromboplastin Time; INR, International Normalized Ratio; APTT, Activated Partial Thromboplastin Time; TT, thrombin Time; HbA1c, Glycated Hemoglobin A1c; GCS, Glasgow Coma Scale; PIV, pan-immune-inflammation value; CHD, Coronary Heart Disease; ICH:intracerebral hemorrhage; BSH, Brainstem Hemorrhage.

**Table 3:** Multivariate logistic analysis of poor prognosis of ICH.

Variables	*β*	S. E	Z	*P*	OR (95%CI)
Intercept	2.47	0.97	2.56	**0.011**	11.88 (1.78 ~ 79.10)
BSH	1.16	0.29	4.00	**<0.001**	3.17 (1.80 ~ 5.59)
APTT	−0.06	0.03	1.99	**0.047**	0.94 (0.89 ~ 0.99)
Bleeding volume	0.06	0.01	4.95	**<0.001**	1.06 (1.04 ~ 1.09)
GCS	−0.28	0.04	6.79	**<0.001**	0.76 (0.70 ~ 0.82)
PIV	0.01	0.00	5.90	**<0.001**	1.01 (1.01 ~ 1.01)

BMI, Body Mass Index; BSH, Brainstem Hemorrhage; APTT, Activated Partial Thromboplastin Time; GCS, Glasgow Coma Scale; PIV, Pan-Immune-Inflammation Value.

**Figure 2:**
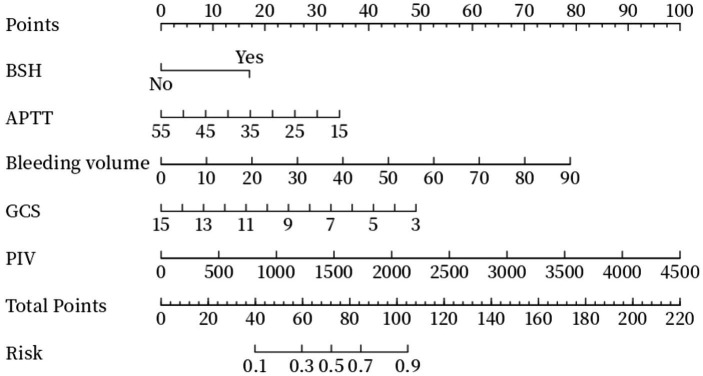
Nomogram plot. Total points are obtained by adding up the points for each of the five indicators (BSH, APTT, bleeding volume, GCS, PIV). Risk is the probability of a poor prognosis occurring as derived from the total points. BSH, Brainstem Hemorrhage; APTT, Activated Partial Thromboplastin Time; GCS, Glasgow Coma Scale; PIV, Pan-Immune-Inflammation Value.

A nomogram for predicting the risk of poor prognosis in patients with ICH was constructed using brainstem hemorrhage, APTT, bleeding volume, GCS score, and PIV as predictor variables (Figure 2).

**Figure 3:**
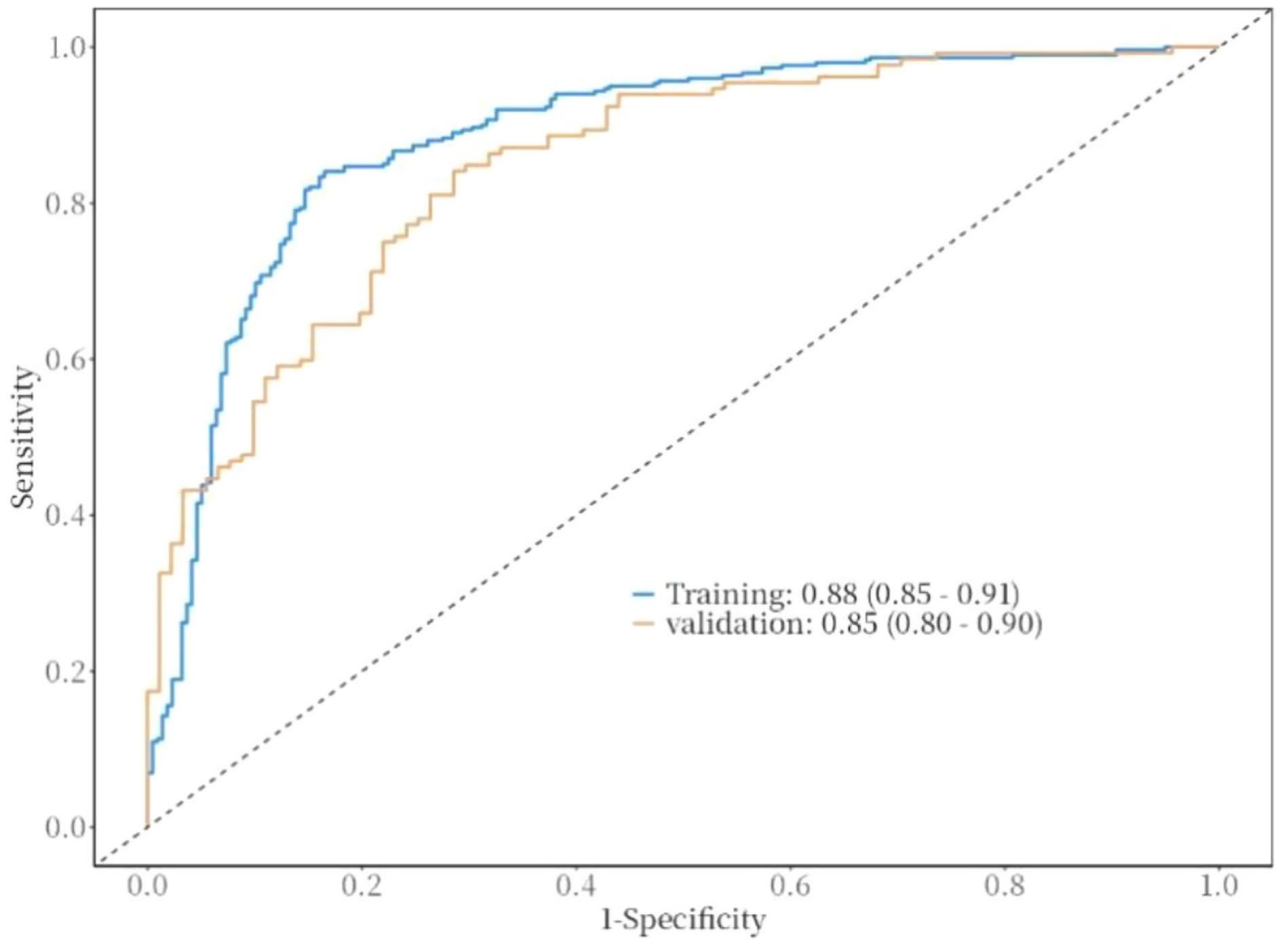
ROC curve of the nomogram for risk of poor prognosis in ICH patients.

**Figure 4:**
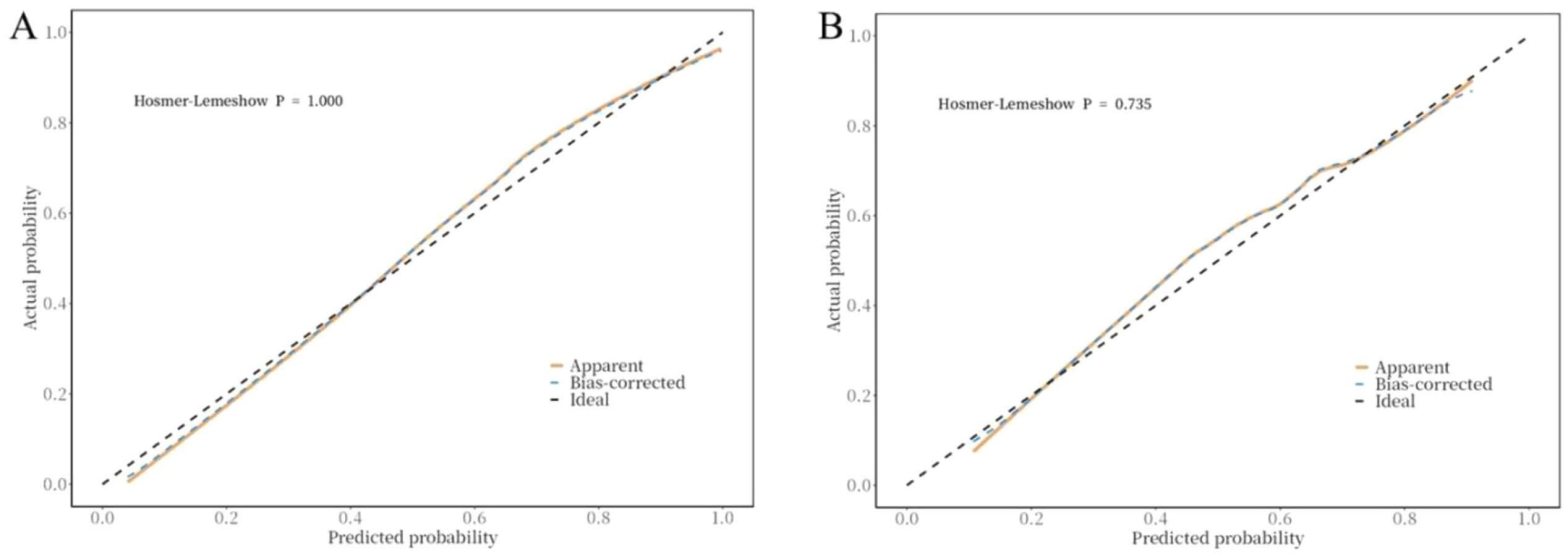
Correction curve of the nomogram for the risk of poor prognosis in ICH patients. **(A)** training cohort, **(B)** validation cohort.

**Figure 5:**
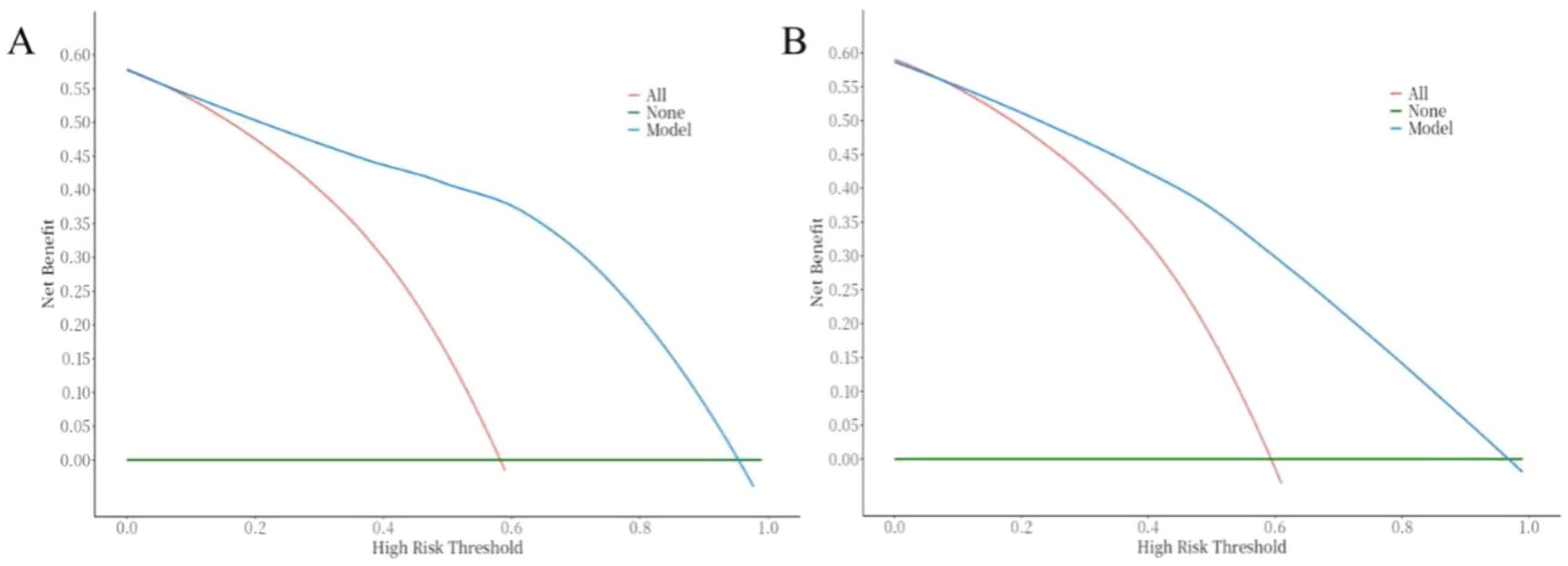
Correction curve of the nomogram for the risk of poor prognosis in ICH patients. **(A)** training cohort, **(B)** validation cohort.

The ROC curve was used to evaluate the discriminative ability of the nomogram for predicting poor prognosis in patients with ICH. The results indicated that the area under the ROC curve (AUC) was 0.88, with a sensitivity of 83% and a specificity of 84%. These findings suggest that the nomogram has good discriminative performance (Figure 3). The Hosmer-Lemeshow goodness-of-fit test demonstrated that the model was well-calibrated. The calibration curves for the training cohort closely approximated the ideal curves, indicating high accuracy of the model (Figure 4). Decision curve analysis (DCA) was employed to assess the clinical utility of the predictive model. The results showed that the nomogram developed in this study provided clinical benefit across a wide range of threshold probabilities in both the training and validation cohorts (Figure 5).

**Figure 6:**
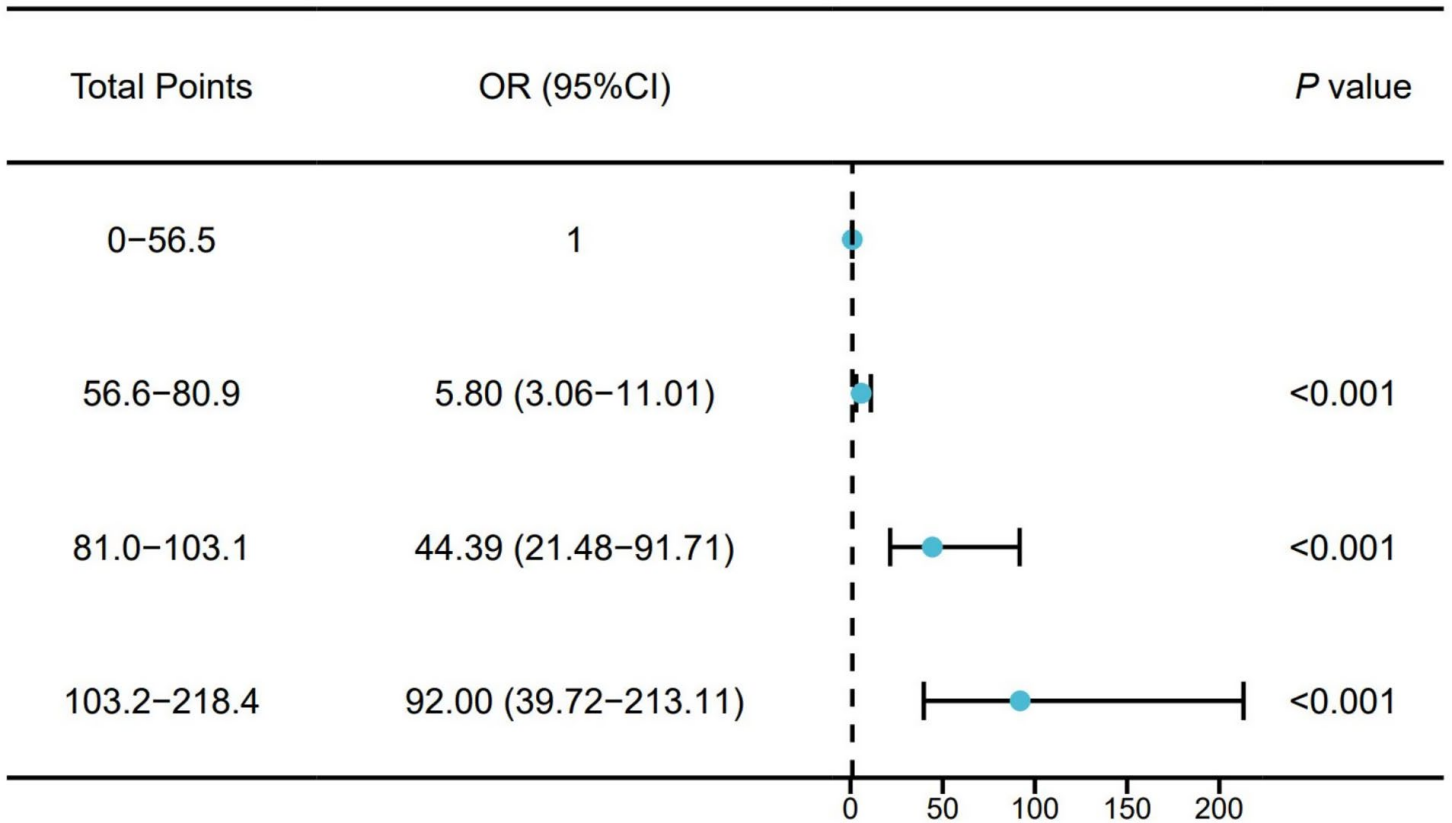
Correlation between different total points intervals and prognosis.

We divided the total points of the training cohort into quartiles and then incorporated them into a Logistic regression model to verify the risk of adverse prognosis in different risk groups. Participants were divided into four groups based on quartiles of the total points. The risk of poor prognosis increased with the total points. Compared with those in the lowest quartile, participants in the highest quartile (total points: 103.2–218.4) had a higher risk of poor prognosis (OR: 92.00, 95% CI: 39.72–213.1) (Figure 6).

## 4 Discussion

Acute ICH is characterized by high lethality and disability rates. A prospective study in China demonstrated that approximately half of the patients had a poor prognosis (15). Early identification of high-risk patients and assessment of their prognosis have been focal points in clinical research. Our study demonstrated that brainstem hemorrhage, decreased APTT, large bleeding volume, low GCS score, and high PIV level are significant risk factors for poor prognosis in ICH. Both primary and secondary injuries following ICH contribute to patient outcomes. Primary brain injury, characterized by the compression of surrounding tissues by the hematoma, leads to decreased cerebral blood flow and subsequent ischemic injury. The severity of this injury is primarily determined by the volume, location, and extent of the hematoma. Consequently, the amount of hemorrhage and bleeding in the brainstem area are critical risk factors for poor prognosis in ICH (16, 17).

As previously demonstrated, prognostic models for ICH built on inflammatory biomarkers can effectively predict short-term outcomes and mortality. Composite inflammatory markers, including NLR, SII, and SIRI, are most widely applied. For example, Zhang et al. (8) showed that both serum NLR and blood glucose levels at admission were closely associated with ICH prognosis. Zhao et al. (18) compared the predictive ability of NLR, PLR, LMR, SII, and SIRI for predicting mortality risks in ICH intensive care unit patients, finding NLR to be a valid predictor for mortality risks in critically ill ICH patients. Yet, no study has specifically investigated the link between PIV and ICH prognosis. In contrast to single markers of inflammation, PIV is a composite index that integrates neutrophil, lymphocyte, platelet, and monocyte counts to quantify systemic inflammation. PIV serves as a novel immune marker that comprehensively represents a patient’s immune and inflammatory status (19). Derived from serum, PIV is easy to obtain and suitable for dynamic monitoring and assessment. It has been utilized in the clinical evaluation of ischemic stroke, subarachnoid hemorrhage, and other diseases (19, 20). Our study confirms that PIV is capable of assessing poor prognosis in patients with ICH and that elevated PIV levels is an independent risk factor for poor prognosis. The inflammatory response is a critical component of secondary brain injury (21). Blood from ruptured vessels flows into the brain tissue, triggering a cascade of immune-inflammatory reactions. This process promotes local infiltration of inflammatory cells and the continuous release of inflammatory mediators, such as cytokines and chemokines, which drive local inflammation, disrupt the BBB, exacerbate cerebral edema, induce neuronal apoptosis, and ultimately lead to irreversible brain damage. Within one to one and a half hours after ICH, microglia, the brain’s resident innate immune cells, are activated by blood components exuded from the hematoma (22). Simultaneously, macrophages migrate from the bloodstream to the central nervous system. Activated microglia/macrophages release a variety of pro-inflammatory factors, chemokines, reactive oxygen species (ROS), and matrix metalloproteinases (MMPs), which promote peripheral inflammatory cell infiltration, disrupt the integrity of the BBB, and induce neuronal apoptosis, ultimately leading to brain damage and neurological dysfunction. Neutrophils, the earliest leukocytes to infiltrate from the peripheral blood into the brain tissue, gradually increase within a few hours after an ICH episode and maintain elevated levels for up to a week. By secreting inflammatory mediators such as tumor necrosis factor-*α*, ROS, and MMP-9, neutrophils disrupt the vascular wall, increasing the permeability of brain capillaries. This process allows peripheral blood inflammatory cells to infiltrate into the brain tissue, generating neurotoxicity, which further exacerbates the disruption of the BBB and the development of cerebral edema (23). Lymphocytes and monocytes are important immune cells in the body and are present in large numbers in the peripheral blood. However, excessive and persistent inflammatory responses following stroke may lead to immune system failure, resulting in systemic immunosuppression, commonly referred to as Stroke-Induced Immunosuppression Syndrome (SIDS) (24, 25). SIDS typically manifests during the acute phase of stroke, primarily due to the body’s stress response. This response leads to over-activation of the sympathetic-hypothalamic–pituitary–adrenal axis, resulting in elevated levels of catecholamines and stress hormones, which contribute to the development of SIDS. SIDS can cause inactivation, depletion, or even apoptosis of lymphocytes and monocytes in the peripheral blood and immune organs, leading to rapid and sustained suppression of cellular immunity. This immune suppression may compromise the body’s ability to resist pathogenic bacteria, increase susceptibility to infections, and lead to severe complications such as post-stroke pneumonia, ultimately resulting in a poor prognosis. Additionally, platelets play a crucial role in the body’s coagulation system. Following ICH, the coagulation balance is disrupted, leading to platelet aggregation, elevated platelet counts, and induced hypercoagulability. These changes can result in adverse events such as deep vein thrombosis in the lower extremities or even pulmonary embolism (26). Activated platelets can exacerbate the systemic immune-inflammatory response by inducing the release of inflammatory cytokines such as adenosine diphosphat and thromboxane A2, thereby causing severe inflammatory reactions (27).

Additionally, our study identified decreased APTT and low GCS score as risk factors for poor prognosis in ICH, findings that are consistent with prior research. Coagulation changes occur within seconds following ICH onset. After an initial period of hypercoagulability, patients typically become hypocoagulable 6–72 h post-ICH. These coagulation disorders can lead to progressive ICH and delayed ICH, thereby increasing the risk of mortality (28, 29). The GCS score correlates with the severity of impaired consciousness in patients with ICH. Lower scores indicate more severe coma and overall condition, which are associated with a higher likelihood of complications during the disease course and, consequently, a higher risk of death. Specifically, the lower the GCS score, the more severe the patient’s coma and condition, and the greater the likelihood of developing complications, thereby implying a higher mortality risk (30).

Based on the aforementioned risk factors, the present study further constructed a nomogram to predict the risk of poor prognosis in patients with ICH. The indicators included in the model are readily accessible in clinical settings and exhibit good discrimination, calibration, and clinical applicability.

This study has several limitations. First, as a retrospective observational study, it only included patients with complete clinical data, which may have introduced selection bias. Second, hematological parameters were assessed only at the time of emergency admission and did not assess subsequent dynamic changes in hematological indices, limiting our ability to dynamically reflect the evolving internal environment during the disease course. Third, all patients were recruited from a single stroke center in East China, which may further contribute to selection bias and limit the generalizability of the findings to broader populations. Regional, racial, lifestyle, and treatment differences may affect the applicability of the results; thus, the conclusions are more representative of the East Chinese population. Further studies involving external validation cohorts across diverse populations are planned to assess the generalizability and predictive accuracy of the proposed nomogram.

## 5 Conclusion

A high PIV level, large bleeding volume, and low GCS score are significant risk factors for poor prognosis in patients with ICH. The nomogram based on these factors demonstrates robust predictive performance.

## Data Availability

The original contributions presented in the study are included in the article/Supplementary material, further inquiries can be directed to the corresponding authors.
