# Supplementary material for: Nomogram based on pan-immune-inflammation value to predict short-term prognosis in spontaneous intracerebral hemorrhage
Source: Front Neurol. 2025 Aug 12;16:1606436. doi: 10.3389/fneur.2025.1606436 (PMC12378056; doi:10.3389/fneur.2025.1606436)
Supplement: Supplementary file 1 [file Data_Sheet_1.pdf]

Supplementary Table 1 Comparison of model performance with single-factor predictive values in the validation cohort

characteristics	AUC	*95%CI*	Sensitivity	Specificity
APTT	0.52	0.46~0.59	0.22	0.89
Bleeding volume	0.75	0.69~0.81	0.92	0.48
GCS	0.73	0.67~0.79	0.87	0.59
PIV	0.71	0.64~0.76	0.52	0.83
nomogram	0.85	0.80~0.90	0.74	0.81

APTT: Activated Partial Thromboplastin Time; GCS: Glasgow Coma Scale; PIV: Pan-Immune-Inflammation Value
